# Effect of Electron-Beam Irradiation on Functional Compounds and Biological Activities in Peanut Shells

**DOI:** 10.3390/molecules28217258

**Published:** 2023-10-25

**Authors:** Narae Han, Jin Young Lee, Mihyang Kim, Jae-Kyung Kim, Yu-Young Lee, Moon Seok Kang, Hyun-Joo Kim

**Affiliations:** 1 Department of Central Area Crop Science, National Institute of Crop Science, Rural Development Administration, Suwon 16613, Republic of Korea; nawrae29@korea.kr (N.H.); jyhello2@korea.kr (J.Y.L.); herbin21c@korea.kr (M.K.); leeyy260@korea.kr (Y.-Y.L.); gr27@korea.kr (M.S.K.); 2 Advanced Radiation Technology Institute, Korea Atomic Energy Research Institute, Jeongeup 56212, Republic of Korea; jkim@kaeri.re.kr

**Keywords:** antioxidant activity, anti-aging, *Arachis hypogaea*, by-product

## Abstract

Peanut shells, rich in antioxidants, remain underutilized due to limited research. The present study investigated the changes in the functional compound content and skin aging-related enzyme inhibitory activities of peanut shells by electron-beam treatment with different sample states and irradiation doses. In addition, phenolic compounds in the peanut shells were identified and quantified using ultra-performance liquid chromatography with ion mobility mass spectrometry–quadrupole time-of-flight and high-performance liquid chromatography with a photodiode array detector, respectively. Total phenolic compound content in solid treatment gradually increased from 110.31 to 189.03 mg gallic acid equivalent/g as the irradiation dose increased. Additionally, electron-beam irradiation significantly increased 5,7-dihydroxychrome, eriodictyol, and luteolin content in the solid treatment compared to the control. However, liquid treatment was less effective in terms of functional compound content compared to the solid treatment. The enhanced functional compound content in the solid treatment clearly augmented the antioxidant activity of the peanut shells irradiated with an electron-beam. Similarly, electron-beam irradiation substantially increased collagenase and elastase inhibitory activities in the solid treatment. Mutagenicity assay confirmed the stability of toxicity associated with the electron-beam irradiation. In conclusion, electron-beam-irradiated peanut shells could serve as an important by-product with potential applications in functional cosmetic materials.

## 1. Introduction

Peanuts (*Arachis hypogaea* L.) are an important crop cultivated worldwide for seed and oil production. Typically regarded as discarded by-products of peanut processing, peanut shells have gained recent recognition for their versatile applications in feedstock, food, and fuel [1,2]. Moreover, peanut shells are utilized as a source of natural antioxidants with high phenolic and flavonoid content, especially luteolin [3,4]. Studies have underscored the beneficial effects of luteolin in maintaining human health by controlling antioxidative stress, aging, and inflammation [5,6]. Agricultural by-products have also been used as natural antioxidants in skin care formulations [7]. Peanut shells are a rich source of functional compounds and have piqued interest as an ingredient in natural cosmetics.

Functional cosmetics are generally classified into whitening, wrinkle improvement, and ultraviolet ray (UV)-protection products. In particular, the main effects of wrinkle-improving cosmetics are known to promote collagen synthesis, strengthen skin elasticity, and promote epidermal metabolism and fibroblast production [8]. Excessive melanin accumulation catalyzed by tyrosinase results in hyperpigmentation disorders including melisma, freckles, and age spots [9]. Additionally, degradation of elastin fiber and collagen complex by elastase and collagenase, respectively, leads to decrease in skin elasticity, flexibility, resiliency, and strength [10]. The development of tyrosinase, collagenase, and elastase inhibitors for use in functional cosmetics is therefore important for the control of whitening, wrinkles, and skin aging. In previous studies, raw materials for functional cosmetics were reported in medicinal plants and herb extract [8,9]; however, the skin-aging-related enzyme (i.g., anti-tyrosinase, anti-collagenase, and anti-elastase) inhibitory activities of food crops and their by-products have not yet been elucidated.

Ionizing radiation, including gamma rays, X-rays, and electron beams, is an effective technology for food preservation [11]. Recently, ionizing radiation technology has been employed to improve bioactive compounds in natural ingredients, as it has the potential to enhance the biological activity of phenolic compounds [12]. Instead of radioisotopes, electron-beam irradiation uses an electrical source to generate ionizing energy. In addition, electron-beam irradiation offers several advantages, such as easy handling, reduced logistics costs, and fewer unexpected adverse effects on the irradiated product [13,14].

In most previous studies, quantitative and qualitative analyses of the phenolic compounds in peanut shell extract were conducted using high-performance liquid chromatography (HPLC) by comparing retention time of peaks with those of standard compounds [15]. To the best of our knowledge, no prior study has extensively characterized the phenolic compound composition of peanut shells using ultra-performance liquid chromatography–ion mobility mass spectrometry–quadrupole time-of-flight (UPLC–IMS–QTOf). Therefore, this study was conducted to evaluate the overall phenolic and flavonoid contents, as well as antioxidant and anti-aging properties of electron-beam-irradiated peanut shells in different sample states (i.e., solid and liquid) and dose levels (i.e., 0, 5, 10, and 20 kGy). This study aimed to determine the polyphenols in peanut shell extracts using UPLC–IMS–QTOf, and changes in response to electron-beam treatment. We established that electron-beam irradiation to be a secure method for enhancing peanut shell biological activity before industrial utilization.

## 2. Results and Discussion

### 2.1. Evaluation of Extract Color and Functional Compound Content in Electron-Beam-Irradiated Peanut Shell

**Figure 1:**
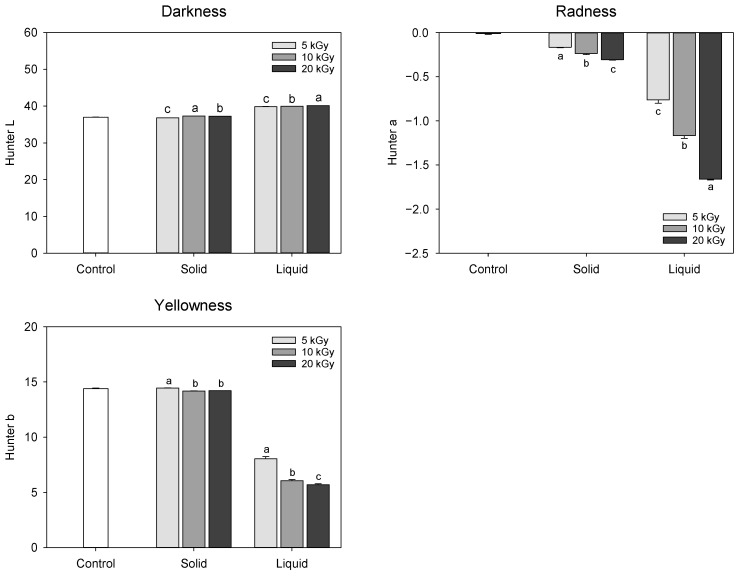
Change in the color of extract obtained from electron-beam-irradiated peanut shell depending on the sample state and irradiation dose level. The values are presented as the mean ± standard deviation of three replicates. Different letters in the same treatment (i.g., solid and liquid) indicate a significant difference between electron-beam dose levels according to Duncan’s multiple range test *p* < 0.05.

The change in the color of extract obtained from electron-beam-irradiated peanut shells is presented in Figure 1. Hunter color ‘L’, ‘a’, and ‘b’ represent the degree of lightness, greenness to redness, and blueness to yellowness, respectively. The lower values of ‘L’, ‘a’, and ‘b’ signify increased darkness, greenness, and blueness, respectively, which are primarily influenced by chemical changes or degradation [16]. In solid treatment, only minimal changes were observed in the Hunter color values, whereas liquid treatment showed a remarkable decline in the ‘a’ and ‘b’ values. In the liquid treatment, the ‘L’ value increased from 37.02 at 0 kGy to 40.10 at 20 kGy, while the ‘a’ and ‘b’ values decreased from −0.01 and −1.66 at 0 kGy to 14.40 and 5.70 at 20 kGy, respectively, resulting in discoloration of the extract color as electron-beam dose level increased. Thus, it was assumed that the electron-beam irradiation of the liquefied peanut shell probably might have a negative influence on the functional compound content or composition. A previous study reported a more pronounced darkening of almonds, hazelnuts, pine nuts, and peanuts when irradiated with an electron-beam; however, these observations were not consistently reproducible [17].

**Figure 2:**
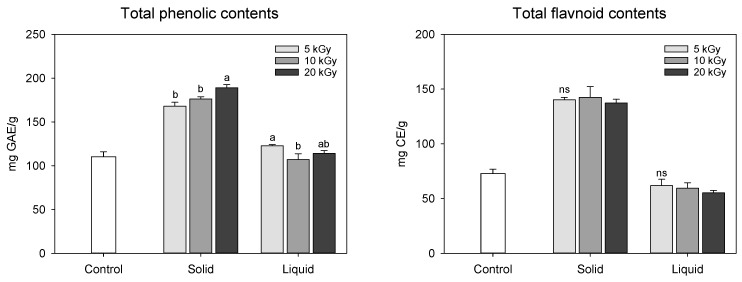
Total phenolic compounds and flavonoid contents in electron-beam-irradiated peanut shell depending on state of the sample and dose level. The values are presented as the mean ± standard deviation of three replicates. Different letters in the same treatment (i.g., solid and liquid) indicate a significant difference between electron-beam dose levels according to Duncan’s multiple range test *p* < 0.05. ns, not significant.

Changes in the functional compound (total phenolic and flavonoid) content in peanut shells treated by an electron-beam in different states of the sample and dose levels are shown in Figure 2. The total phenolic and flavonoid contents in solid treatment was dramatically increased when treated with an electron-beam from 110.31 mg gallic acid equivalent (GAE)/g at 0 kGy to 189.03 mg GAE/g at 20 kGy; however, the liquid treatment was less effective than the solid treatment (107.07–122.83 mg GAE/g). As the irradiation dose increased, the total phenolic compound content augmented during the solid treatment. Electron-beam irradiation also enhanced flavonoid concentration in solid treatment compared to the control to a value of 72.84 mg catechin equivalent (CE)/g, but there was no statistical difference between the dose levels (137.28–142.40 mg CE/g). Han et al. [15] reported that the total phenolic and flavonoid contents in peanut shell extract were 253.94 mg GAE/g and 111.74 mg CE/g, respectively, which were higher than those observed in this study. These differences may be attributed to variations in experimental methods, cultivation environments, and climatic conditions. Previous studies have conducted thermal (i.e., boiling and roasting) and/or non-thermal treatments (i.e., gamma and far-infrared radiation) to enhance the functional compound content in nuts, such as peanuts, hazelnuts, pine nuts, and almonds [1,17,18]. Zhang et al. [19] reported that the total flavonoid content of peanut skin increased following ozone treatment. Recently, we reported that atmospheric pressure plasma treatment increases the total phenolic and flavonoid content in peanut shells [3]. In this study, it was postulated that the increase in the functional compound content in the solid treatment may be attributed to cell wall modification and/or decomposition of the chemical bonds of the polyphenolic compounds induced by electron-beam irradiation. In addition, it was also assumed that electron-beam irradiation of the liquid-type sample might cause an excessive breakdown of phenolic compounds in the peanut shell extracts, resulting in decreased yellow color and flavonoid content.

### 2.2. Evaluation of Biological Activities in Electron-Beam-Irradiated Peanut Shell

**Figure 3:**
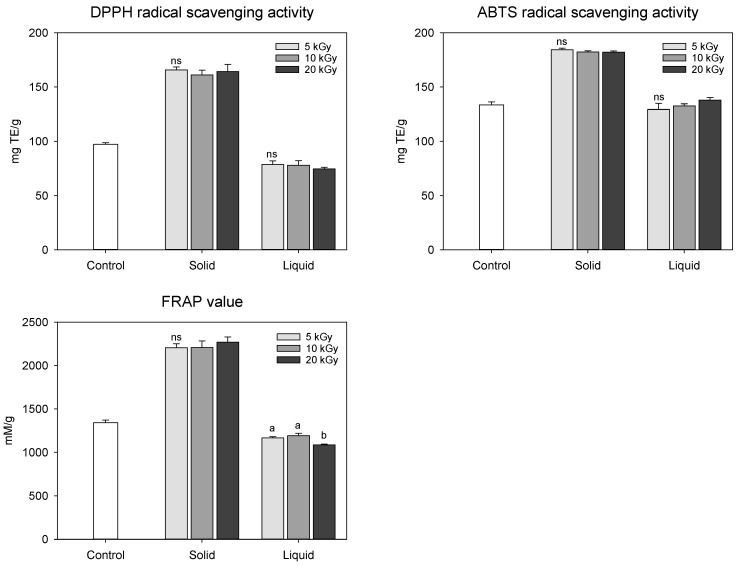
Antioxidant activities in electron-beam-irradiated peanut shell depending on the sample state and irradiation dose level. The values are presented as the mean ± standard deviation of three replicates. Different letters in the same treatment (i.g., solid and liquid) indicate a significant difference between electron-beam dose levels according to Duncan’s multiple range test *p* < 0.05. ns, not significant.

Antioxidant activity is a typical indicator of various polyphenolic compounds present in peanuts. Thus, the antioxidant activities of polyphenolic compounds can be determined based on their free radical such as 2,2-diphenyl-1-picrylhydrazyl (DPPH) and 2,2′-azino-bis(3-ethylbenzothiazoline-6-sulfonic acid) (ABTS) scavenging activities and reductive potential by ferric ion reducing antioxidant potential (FRAP). The antioxidant activities of irradiated peanut shells were affected by the state of the sample (Figure 3). The solid treatment exhibited higher antioxidant activity than the control and liquid treatments. Hwang et al. [11] observed that electron-beam irradiation increased the antioxidant activity of mugwort extracts as the irradiation dose increased from 2 to 10 kGy. However, in our study, electron-beam irradiation at doses between 5 and 20 kGy showed no statistical difference in antioxidant activities. This could be attributed to the fact that there was no notable variation in the functional compound content, which was highly correlated with antioxidant activity. DPPH, ABTS, and FRAP activities of the peanut shells were strongly correlated with the total phenolic (*r*_DPPH_ = 0.946, *r*_ABTS_ = 0.952, and *r*_FRAP_ = 0.956) and flavonoid (*r*_DPPH_ = 0.993, *r*_ABTS_ = 0.976, and *r*_FRAP_ = 0.986) contents (*p* < 0.001). Hwang et al. [11] also demonstrated that irradiation treatment modifies the cell wall and facilitates the emission of extractable substances such as polyphenols, resulting in increased antioxidant activity. Our findings were consistent with these results.

**Figure 4:**
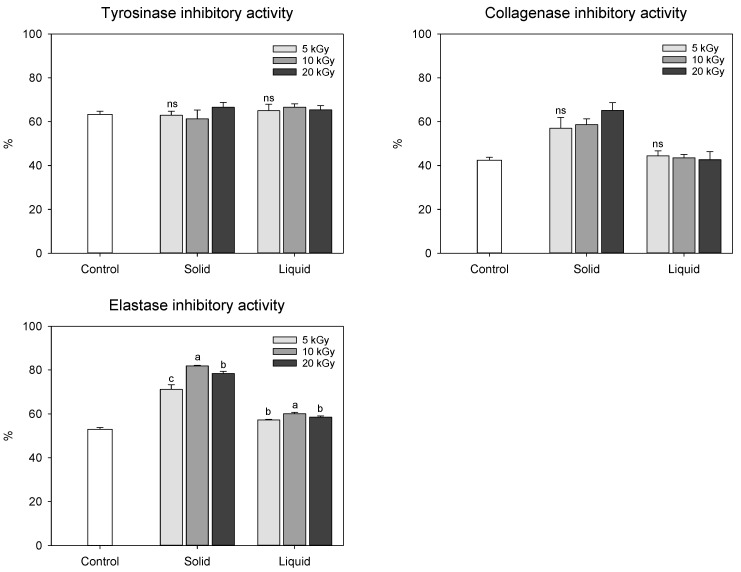
Skin aging-related enzyme inhibition effect of electron-beam-irradiated peanut shell depending on the state of the sample and dose level. The values are presented as the mean ± standard deviation of three replicates. Different letters in the same treatment (i.g., solid and liquid) indicate a significant difference between electron-beam dose levels according to Duncan’s multiple range test *p* < 0.05. ns, not significant.

We also evaluated skin anti-aging activities of electron-beam-irradiated peanut shells, given their reputation as a valuable source of antioxidants (Figure 4). A tyrosinase activity assay was primarily implemented to assess skin whitening, and collagenase and elastase inhibitory activities are usually measured to determine wrinkle improvement [10,20]. Tyrosinase inhibitory activity in peanut shells showed no statistical difference in the range of 61.35–66.63%, regardless of the state of the sample or dose levels. Collagenase inhibitory activity in the solid treatment (50.00–65.12%) was significantly enhanced by electron-beam irradiation compared to that in the control (42.42%), whereas no statistical difference was observed between the control and liquid treatments (42.62–44.45%). Similar to the results of the collagenase inhibition assay, the most potent anti-elastase effect was observed in the solid treatment, especially at a dose of 10 kGy. The enhanced anti-collagenase and anti-elastase activities in the solid treatment were likely influenced by the increase in phenolic compound content induced by the irradiated electron-beam.

### 2.3. Identification of Phenolic Compounds in Peanut Shell Using Ultra-Performance Liquid Chromatography–Ion Mobility Mass Spectrometry–Quadrupole Time-of-Flight (UPLC–IMS–QTOf) and Relative Quantification of Major Phenolic Compounds in Electron-Beam-Irradiated Peanut Shell Using High-Performance Liquid Chromatography Coupled with Photodiode Arrary Detector (HPLC–PDA)

**Figure 5:**
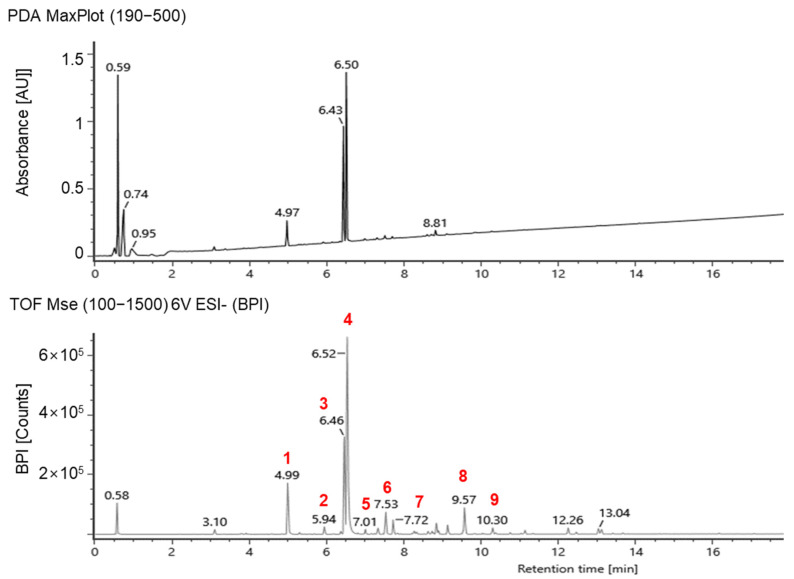
A representative UPLC–IMS–QTOf chromatogram of peanut shell extracts. The compound codes (1–9) are provided in Table 1.

Phenolic compounds in the peanut shells were identified using UPLC–IMS–QTOf, and the chromatograms are shown in Figure 5. Nine compounds were tentatively identified in the peanut shells from the polyphenol database, with most of them belonging to flavonoids and flavonoid subclasses (Table 1). Among the phenolic compounds, 5,7-dihydroxychromone, eriodictyol, and luteolin were the major compounds, consistent with previous studies [15,21]. One phenolic acid, 5-hydroxyferulic acid, was identified in peanut shells; however, the peaks were lower than the flavonoid peaks detected. Compounds 6 and 7 showed the same [M−H]^−^ at *m*/*z* 299.0561, suggesting the possibility of being an isomeric pair; they were tentatively identified as chrysoeriol and paratensein, respectively. 

**Figure 6:**
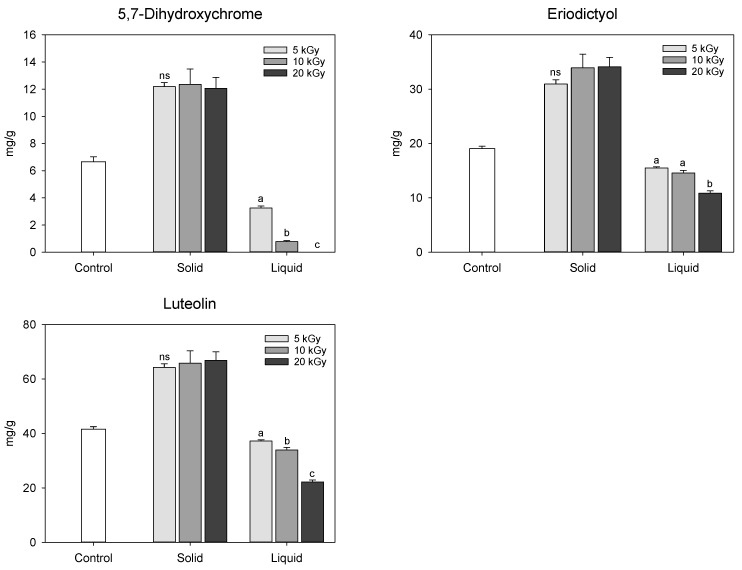
Content of the major phenolic compounds in electron-beam-irradiated peanut shell depending on the state of the sample and irradiation dose level. The values are presented as the mean ± standard deviation of three replicates. Different letters in the same treatment (i.g., solid and liquid) indicate a significant difference between electron-beam dose levels according to Duncan’s multiple range test *p* < 0.05. ns, not significant.

In this study, the UPLC chromatogram confirmed that the composition of the extracted phenolic compounds in electron-beam-irradiated peanut shells was similar to that of the control, but the relative peak area differed depending on the treatment. The 5-hydroxyferulic acid, apigenin, chrysoeriol, pratensein, 8-prenyl luteolin, and caflanone, identified by UPLC–IMS–QTOf analysis, were not detected in high-performance liquid chromatography (HPLC) coupled with photodiode array (PDA) chromatogram, possibly because of their low concentrations. Thus, except for these six compounds, the relative quantification of 5,7-dihydroxichromone, eriodictyol, and luteolin in the electron-beam-irradiated peanut shells was performed using HPLC-PDA by comparing them with their individual standards (Figure 6). The method validation showed linearity, with a correlation coefficient of 0.999. Among the three chemicals, luteolin was predominant in the control at 41.56 mg/g, followed by eriodictyol (19.08 mg/g) and 5,7-dihydroxychrome (6.66 mg/g). Regardless of the dose levels, the contents of these three chemicals significantly increased in the solid treatment following electron-beam irradiation compared with the control. Conversely, all the contents decreased to approximately 30.3–100% in the liquid treatment as the irradiation dose increased. As previously mentioned, electron-beam irradiation at doses of 5–20 kGy did not significantly affect the phenolic compound content in peanut shells. Thus, additional studies are needed to compare the changes in phenolic compounds in peanut shells after electron-beam treatment at various doses. Qiu et al. [21] identified antioxidants in peanut shell as 5,7-dihydroxhchromone, eriodictyol, and luteolin with lower contents of 0.95, 0.92, 2.36 mg/g, respectively, compared to the findings in this study. The variation in the content of these compounds reported in the literature is probably due to differences in sample genotypes and extraction methods.

### 2.4. Mutagenicity Assay

Mutagenicity is evaluated as positive when the number of revertant colonies in the treatment is more than twice that of the control, showing a dose-dependent trend [22]. As shown in Table 2, the number of revertant colonies in the positive control with TA98 (−S9), TA98 (+S9), TA100 (−S9), and TA100 (+S9) strains was approximately 11.5, 12.2, 2.1, and 3.6 times higher than that of negative control, respectively. However, there was no difference between the sample and the positive control at concentrations up to 4 mg/plate, regardless of electron-beam irradiation. 

## 3. Materials and Methods

### 3.1. Reagents and Standards

All standard chemicals, enzymes, substrates, buffers, and positive controls used in this study were purchased from Sigma Aldrich (St. Louis, MO, USA). HPLC-grade water, ethanol, and acetonitrile were purchased from J.T. Baker, Inc. (Phillipsburg, NJ, USA). Distilled water was obtained using a Milli-Q Advantage A10 water-purification system (Merck Millipore, Billerica, MA, USA). 

### 3.2. Plant Materials and Sample Preparation

Peanuts (*Arachis hypogaea* cv. Sinpalkwang) were sourced from a peanut farmhouse (Gochang, Republic of Korea). The peanuts were washed with tap water, and the peanut shells and kernels were separated. The resulting peanut shells (solid treatment) and peanut shell extracts (liquid treatment) were used in the experiments. Peanut shell extracts were prepared as previously described by Han et al. [15] with slight modifications. The ground peanut shell (4 g) was mixed with 40 mL of 100% ethanol and incubated with shaking at 25 °C for 24 h. The mixture was centrifuged (CR22N; Eppendorf Himac Technologies Co., Ltd., Ibaraki, Japan) at 10,000× *g* for 20 min. The supernatant was then collected and used for further experiments. 

### 3.3. Electron-Beam Irradiation

Two states of the sample (i.e., raw materials for the solid state and extracts for the liquid state) were placed in a 50 mL tube with a screw cap at room temperature and exposed to four absorbed doses, i.e., 0 (non-irradiated), 5, 10, and 20 kGy, with electron-beam sources. A UELV-10-10S electron-beam accelerator (10 MeV, 0.2 mA, Moscow, Russia) was used at the Advanced Radiation Technology Institute, Korea Atomic Energy Research Institute (Jeongeup, Republic of Korea). The radiation source was set at a rate of 10 kGy/h. The absorbed doses were evaluated with alanine dosimeters (diameter 5 mm, Bruker Instruments, Bremen, Germany), and the actual dose was within ±5% of the target dose. 

Extracts of the electron-beam-treated peanut shells (solid treatment) were prepared as described in Section 3.2. The extracts of irradiated peanut shell (solid treatment) and irradiated peanut shell extracts (liquid treatment) were evaporated using a rotary evaporator (SB-1200, EYELA Co., Ltd., Tokyo, Japan). The samples were redissolved in 100% ethanol for UPLC analysis and in dimethyl sulfoxide (DMSO) for functional compound, biological activity, and mutagenicity assays, following previous studies [3,10]. 

### 3.4. Determination of Extract Color

The color of the extracts was evaluated using a chromameter (CM-3500d; Minolta, Tokyo, Japan) with three replicates. The measurements are recorded in L (darkness–whiteness), a (greenness–redness), and b (blueness–yellowness) spectra. 

### 3.5. Determination of Functional Compound Contents

The electron-beam-irradiated peanut shells were quantified for total phenolic and flavonoid contents using the modified Folin–Ciocalteu [23] and aluminum chloride methods [24], respectively. Total phenolic and flavonoid contents were expressed as mg GAE/g extract and mg CE/g extract, respectively. 

### 3.6. Evaluation of Antioxidant Activities

The radical scavenging activities of DPPH, ABTS, and FRAP were analyzed to evaluate the antioxidant activity of the electron-beam-irradiated peanut shell extracts, as described in our previous study [3]. These assessments were expressed as mg Trolox equivalent (TE)/g extract, and the FRAP activity was expressed as mM/g extract. 

### 3.7. Evaluation of Biological Activities

Tyrosinase, collagenase, and elastase inhibitory activities were analyzed to evaluate the anti-aging potential of the extracts following the enzymatic method described by Han et al. [15]. To evaluate tyrosinase inhibitory activity, the dopachrome method with L-3,4-dihydroxyphenylalanine as the substrate was used. Collagenase inhibitory activity was evaluated using a spectrofluorometric method with metalloproteinase-2 as the substrate. In addition, elastase inhibitory activity was evaluated by detecting released *p*-nitroaniline from N-succinyl-Ala-Ala-Ala-*p*-nitroanilide by elastase. Kojic acid, chlorhexidine, and elastatinal were used as positive controls for tyrosinase, collagenase, and elastase inhibition assays, respectively. The inhibition (%) was calculated as follows:

Inhibitory activity (%) = [1 − {(A_sample_ − A_sample blank_)/(A_control_ − A_control blank_)}] × 100,

where A_sample_ is the absorbance or fluorescence of a mixture consisting of a sample, enzyme, and substrate; A_sample blank_ is the absorbance or fluorescence of a mixture without the enzyme; A_control_ is the absorbance or fluorescence of a mixture without the sample; and A_control blank_ is the absorbance or fluorescence of a mixture without the sample or enzyme. The samples were tested at concentrations of 1, 0.1, and 1 mg/mL for tyrosinase, collagenase, and elastase inhibitory activities, respectively.

### 3.8. Identification and Relative Quantification of Phenolic Compounds in Peanut Shell Using UPLC–IMS–QTOf and HPLC-PDA

Phenolic compounds in peanut shells were identified using an ACQUITY UPLC equipped with IMS–QTOf via electrospray ionization (ESI) (Vion IMS, Waters, Milford, MA, USA). The chemicals were separated using an ACQITY UPLC BEH C18 column (2.1 × 100 mm, 1.7 μm particle size; Waters). The mobile phases were water with 0.1% formic acid (A) and acetonitrile (B) with 0.1% formic acid, which were applied using the gradient method (5% B for 0–1 min, 5–100% B for 1–20 min, 100% B for 20–22.5 min, and 100–5% B for 22.5–25 min) at 1 mL/min. To identify all possible phenolic compounds, total ion spectra were collected over a mass range of *m/z* 100–1500 in the negative mode. The gas temperature and gas flow rate was 350 °C and 800 L/min, respectively. The ESI conditions were a capillary voltage of −2300 V and collision voltage of 40 V. The accurate mass of the phenolic compounds was calculated based on their molecular formula in the database, and the compounds were identified by comparing their observed accurate masses with the calculated theoretical masses. 

The major phenolic compounds in the peanut shells identified by UPLC–IMS–QTOf, 5,7-dihydroxychrome, eriodictyol, and luteolin were quantified using a Chromaster HPLC (Hitachi Ltd., Tokyo, Japan) coupled with a PDA detector. The stationary mobile phase used in the UPLC–IMS–QTOf detection was used for relative quantification. The retention times of the peaks in the HPLC chromatograms were compared with those of commercial standards. Quantification was performed using three different standard curves, and the concentration was expressed as mg/g of the extract. 

### 3.9. Mutagenicity Assay

A higher dose (20 kGy) was used for the mutagenicity assay, and the samples were tested at concentrations of 4, 2, 1, 0.5, and 0.25 mg/plates. A *Salmonella* mutagenicity assay was performed as described by Jo et al. [25]. *Salmonella typhimurium* strains TA98 and TA100 were purchased from Molecular Toxicology, Inc. (Boone, NC, USA). The strains were inoculated on nutrient broth No. 2 (Oxoid Co., Ltd., Hampshire, England) at 37 °C for 10 h. A 5% S9 Mix. (Lot No. 0042101; Oriental Yeast Co., Ltd., Tokyo, Japan) mixed with a cofactor (Lot No. 999902; Wako Co., Tokyo, Japan) was prepared at a concentration of 0.5 mL/plate. The sample (100 μL), 500 μL of S9 Mix (+S9) or sterilized water (−S9), and 100 μL of TA98 or TA100 strains were mixed with 2 mL of tap agar containing histidine-biotin. The mixture was then poured directly into minimal glucose agar. Sodium azide (SA), 4-nitroquinoline-1-oxide (4-NQO), and 2-aminoanthracene (2-AA) served as positive controls, and DMSO was used as a negative control. The plate was incubated at 37 °C for 48 h, and the number of revertant colonies was counted. All experiments were performed in triplicates. Thus, there were 2 samples (non-irradiated and 20 kGy) × 5 concentrations (4, 2, 1, 0.5, and 0.25 mg/plate) × 2 strains (TA98 and TA100) × 2 metabolic activators (+S9 and –S9) × 3 replicates = 120 plates for mutagenicity assay. 

### 3.10. Statistical Analysis

All data were presented as the average of the values of the replicates (*n* = 3), with standard deviation using SigmaPlot software (version 14.0; Systat Software, San Jose, CA, USA). Irradiation dose (5, 10, and 15 kGy) differences in the raw materials and extracts were evaluated using Tukey’s multiple range test at *p* < 0.05, using SPSS statistical software (version 18.0, SPSS Inc., Chicago, IL, USA).

## 4. Conclusions

In this study, nine phenolic compounds in peanut shells were identified using UPLC–IMS–QTOf, and the changes in the major compounds detected in the peanut shells treated in electron-beam irradiation were evaluated. Solids treatment improved the antioxidant properties and skin aging-related enzyme inhibitory capacities of peanut shells by increasing their polyphenolic content. The stability of toxicity related to electron-beam irradiation was confirmed by a mutagenicity assay, suggesting that the electron-beam irradiation could be a safe technique to boost the biological activity of peanut shells prior to their use in industrial applications. Further studies are needed to confirm the optimal irradiation dose to maximize the biological activity of peanut shells. 

## Figures and Tables

**Table 1:** Identified phytochemical compounds in peanut shell extracts by using UPLC-coupled to QTOf MS/MS.

Peak No.	Tentative Identification	Rt (min)	Molecular Formula	Theoretical (*m*/*z*)	Error (ppm)
1	5,7-Dihydroxychromone	4.99	C_9_H_6_O_4_	177.0193	1.19
2	5-Hydroxyferulic acid	5.94	C_10_H_10_O_5_	209.045547	2.32
3	Eriodictyol	6.46	C_15_H_12_O_6_	287.0561	0.91
4	Luteolin	6.52	C_15_H_10_O_6_	285.0406	3.60
5	Apigenin	7.01	C_15_H_10_O_5_	269.0455	1.72
6	Chrysoeriol	7.53	C_16_H_12_O_6_	299.0561	0.36
7	Pratensein	7.72	C_16_H_12_O_6_	299.0561	0.36
8	8-Prenyl luteolin	9.57	C_20_H_18_O_6_	353.1031	1.73
9	Caflanone	10.30	C_21_H_2_0O_6_	367.1187	0.96

**Table 2:** Mutagenicity assay of the irradiated peanut shell (0 and 20 kGy) with (+) and without (−) metabolic activation (S9) using *Salmonella typhimurium* TA98 and TA100 bacterial strains.

Sample	Concentration (mg/plate)	Number of Revertant Colonies (His+) per Plate			
		TA98 (−S9)	TA98 (+S9)	TA100 (−S9)	TA100 (+S9)
0 kGy	4	23 ± 6	44 ± 4	299 ± 16	277 ± 17
(non-treated)	2	33 ± 4	52 ± 8	243 ± 24	230 ± 23
	1	25 ± 8	30 ± 6	202 ± 13	223 ± 14
	0.5	23 ± 6	33 ± 5	241 ± 8	271 ± 10
	0.25	19 ± 4	51 ± 4	218 ± 16	210 ± 10
20 kGy	4	29 ± 7	48 ± 5	170 ± 14	167 ± 14
	2	32 ± 4	42 ± 8	241 ± 15	228 ± 22
	1	28 ± 6	44 ± 3	213 ± 25	233 ± 23
	0.5	26 ± 3	41 ± 8	241 ± 24	245 ± 21
	0.25	29 ± 4	44 ± 6	256 ± 7	244 ± 22
Negative control	DMSO	28 ± 8	41 ± 9	188 ± 19	202 ± 8
Positive control	4-NQO	323 ± 6			
	2-AA		500 ± 60		
	SA			403 ± 31	
	2-AA				737 ± 23

All data are presented as the mean ± standard deviation of three replicates. DMSO, dimethyl sulfoxide; 4-NQO, 4-mitroquinoline-1-oxide; 2-AA, 2-aminoanthracene; SA, sodium azide.

## Data Availability

Data are contained within the article.
